# Efficacy of CD46-targeting chimeric Ad5/35 adenoviral gene therapy for colorectal cancers

**DOI:** 10.18632/oncotarget.9427

**Published:** 2016-05-18

**Authors:** Young-Suk Cho, Manh-Hung Do, Se-Young Kwon, Changjong Moon, Kwonseop Kim, Keesook Lee, Sang-Jin Lee, Silvio Hemmi, Young-Eun Joo, Min Soo Kim, Chaeyong Jung

**Affiliations:** ^1^ Department of Anatomy, Chonnam National University Medical School, Gwangju, Korea; ^2^ College of Veterinary Medicine, Chonnam National University, Gwangju, Korea; ^3^ College of Pharmacy, Chonnam National University, Gwangju, Korea; ^4^ Hormone Research Center, School of Biological Sciences and Technology, Chonnam National University, Gwangju, Korea; ^5^ Genitourinary Cancer Branch, Research Institute of National Cancer Center, Goyang, Gyeonggi-do, Korea; ^6^ Institute of Molecular Life Sciences, University of Zurich, Zurich, Switzerland; ^7^ Department of Internal Medicine, Chonnam National University Medical School, Gwangju, Korea; ^8^ Department of Statistics, College of Natural Sciences, Chonnam National University, Gwangju, Korea

**Keywords:** CD46, adenovirus, gene therapy, colorectal cancer

## Abstract

CD46 is a complement inhibitor membrane cofactor which also acts as a receptor for various microbes, including species B adenoviruses (Ads). While most Ad gene therapy vectors are derived from species C and infect cells through coxsackie-adenovirus receptor (CAR), CAR expression is downregulated in many cancer cells, resulting inefficient Ad-based therapeutics. Despite a limited knowledge on the expression status of many cancer cells, an increasing number of cancer gene therapy studies include fiber-modified Ad vectors redirected to the more ubiquitously expressed CD46. Since our finding from tumor microarray indicate that CD46 was overexpressed in cancers of the prostate and colon, fiber chimeric Ad5/35 vectors that have infection tropism for CD46 were employed to demonstrate its efficacy in colorectal cancers (CRC). CD46-overexpressed cells showed a significantly higher response to Ad5/35-GFP and to Ad5/35-tk/GCV. While CRC cells express variable levels of CD46, CD46 expression was positively correlated with Ad5/35-mediated GFP fluorescence and accordingly its cell killing. Injection of Ad5/35-tk/GCV caused much greater tumor-suppression in mice bearing CD46-overexpressed cancer xenograft compared to mock group. Analysis of CRC samples revealed that patients with positive CD46 expression had a higher survival rate (p=0.031), carried tumors that were well-differentiated, but less invasive and metastatic, and with a low T stage (all p<0.05). Taken together, our study demonstrated that species B-based adenoviral gene therapy is a suitable approach for generally CD46-overexpressed CRC but would require careful consideration preceding CD46 analysis and categorizing CRC patients.

## INTRODUCTION

Colorectal cancers (CRC) are the third most common cancer in the United States, with an estimated 132,700 new cases in 2015 [1]. Metastatic CRC is the second leading cause of death from cancer with an estimated 49,700 deaths in 2015, largely due to the poor clinical response of tumors to conventional chemotherapeutics. Application of adenoviral gene therapy has been receiving interest to overcome poor therapeutic efficacy of conventional cancer treatment [2, 3].

Adenovirus (Ad) has been attractive tool for cancer gene therapy as a result of its high transduction efficiency and a large quantity of information acquired from many clinical trials. Its efficiency for cancer gene therapy, however, has been limited mainly due to the lack of cancer cell targeting, presence of neutralizing antibodies, and hepatotoxicity [4–7]. So far, 57 serotypes of human Ads have been identified, which are divided into species A to G. The most commonly used Ad vectors are based on species C Ad5, which primarily binds to the coxsackie-adenovirus receptor (CAR) [8]. Many tumor cells, however, express relatively low levels of CAR, which essentially renders the tumor cell resistant to Ad5 infection [4, 5]. Moreover, as most adults are immune to Ad5, a high dose is required for effective therapy [6]. Species B adenoviruses are subdivided into B1 and B2. The B1 subspecies includes Ad3, Ad7, Ad16, Ad21, and Ad50 whereas the B2 subspecies includes Ad11, Ad14, Ad34, and Ad35. Viral tropism of species B to cancer cells, dendritic cells, and hematopoietic stem cells is broader than that of species C [9, 10]. Species B adenoviruses bind to different cell surface receptors than other members of the Ad family. These include CD46 for Ad3/11/35 [11–13] and desmoglein 2 (Dsg2) for Ad3/7/11/14 [14, 15].

CD46 is a type I membrane protein that plays an important inhibitory role in complement-dependent cytotoxicity (CDC) [16]. Human cells most commonly express 3 types of complement restriction factors (also called membrane cofactor proteins): CD55, CD46, and CD59. CD46 protects host cells from CDC by binding to C3b, inactivating C3b and C4b, and preventing subsequent degradation by the plasma serine protease [17]. CD46 also acts as a receptor for numerous pathogenic microbes, including group B adenoviruses [18]. CD46 is also overexpressed in some human cancers, including lymphomas, breast cancers, ovarian cancers, hepatocellular carcinomas [19–22], thus protecting cancer cells from the complement system. Therefore, group B Ads are attractive gene therapy vectors since they can overcome the limitations in tumor transduction efficacy through utilizing more ubiquitously expressed CD46. More recently, there is an increasing number of studies using chimeric adenoviruses containing the Ad35 fiber knob in the context of the Ad5 backbone to enhance the antitumor capability. In fact, these chimeric vectors with modified fiber knobs have been reported to improve gene transduction efficiency [6, 23–29].

Despite the increasing number of cancer therapeutic approaches using these chimeric adenoviral vectors, the detailed expression status of CD46 as a viral target in human cancers remains to be elucidated. In this study, we first investigated the expression levels of CD46 in various tumors using tissue microarrays, demonstrating that CD46 overexpression was prevalent in prostate cancers (PCa) and colorectal cancers (CRC). We then focused on CD46 expression in selected CRC patient samples to determine the correlation between CD46 and clinical outcomes. Several CRC cell lines characterized for their CD46 expression levels were further analyzed for viral transduction and viral cell toxicity using Ad5/35-GFP and Ad5/35-tk, respectively. The tumor-specific killing effect mediated by Ad5/35-tk in combination with ganciclovir (GCV) was also investigated in xenograft nude mouse models.

## RESULTS

### CD46 is overexpressed in cancers of the colorectum and prostate

In order to profile the expression level of CD46 in human cancers, tumor tissue microarray slides were stained with anti-CD46 antibodies. Within the pool of normal tissues including brain, peripheral nerve, adrenal gland, thyroid gland, bone marrow, spleen, lymph node, lung, heart, skeletal muscle, salivary gland, pancreas, stomach, small intestine, colon, mesothelium, kidney, ovary, prostate, testis, and uterus, immunoreactivity was observed only in the liver (data not shown). In tumor tissues, CD46 was most prevalently expressed in cancers of the colon and prostate, in which more than 35 % of the tumors overexpressed CD46, while CD46 expression was less than 11% in other tumors (Table 1 and Supplementary Figure S1). CD46 expression in both prostate and colon cancers is significantly higher than tumors from other origins (Q=56.85 with 7df, p<0.0001 Cochran-Mantel-Haenszel statistics based on Row Mean Scores Differ). Considering that the actual tumor cells represent only a small fraction within the tumor microarray, CD46 was quite highly expressed in both PCa and CRC. CD46 has been implicated in the development and progression of several cancer types. Elevated expression of CD46 has been observed in medulloblastomas, breast cancers, and CRCs [30–32]. Although the expression level of CD46 was significantly higher in CRC tissues compared with the adjacent normal colon tissues, CD46 expression was not associated with colon cancer progression [31]. Due to the on hand availability of CRC tissues and to difficulties in evaluating the notoriously heterogeneous population of PCa, we limited our further studies to CRC.

**Table 1:** Score of CD46 expression in human tumors

Tumor types	Score of CD46 expression*						
	0	1	2	3	0-1**	2-3**	total
Brain tumor	21	0	0	0	21	0 (0 %)	21
Melanoma	17	1	1	1	18	2 (10 %)	20
Ovarian cancer	25	1	1	0	26	1 (3.7 %)	27
Lymphoma	39	1	2	0	40	2 (4.8 %)	42
Lung cancer	41	9	4	2	50	6 (10.7 %)	56
Breast cancer	34	1	1	2	35	3 (7.9 %)	38
Colorectal cancer	24	7	13	4	31	17 (35.4 %)#	48
Prostate cancer	21	12	17	2	33	19 (36.5 %)#	52

* CD46 expression was scored as followed: 0, no; 1, low and focal; 2, moderate; 3, high

** Scores were categorized by 0-1 as normal expression and 2-3 as overexpression

# CD46 expression is significantly higher than tumors from other origins (Q=56.85 with 7df, p<0.0001 Cochran-Mantel-Haenszel statistics based on Row Mean Scores Differ)

### Redirection of Ad5 vector to CD46 with Ad35 fiber knob enhances the transduction

**Figure 1:**
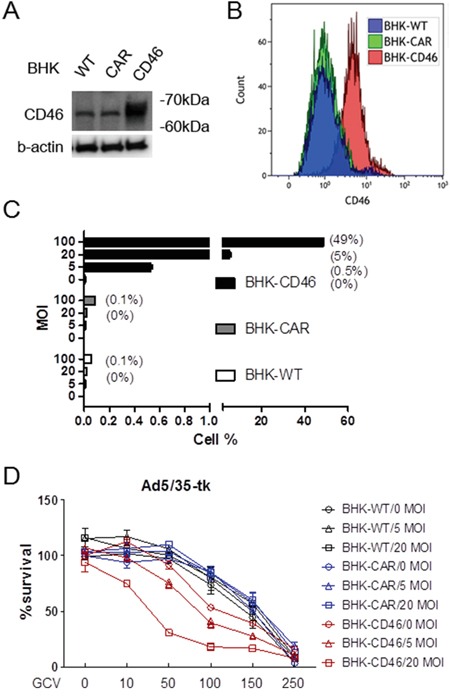
Gene transduction efficacy of Ad5/35 is enhanced in CD46-expressing cells **A.** Western blot analysis of CD46 expression in parental rodent BHK cells or BHK-CAR and BHK-CD46 cells which ectopically express the Ad receptors CAR and CD46, respectively. **B.** Flow cytometric analysis of CD46 expression in BHK, BHK-CAR and BHK-CD46 cells. **C.** Transduction analysis of BHK, BHK-CAR and BHK-CD46 cells using different doses of Ad5/35-GFP. Cells were analyzed for GFP reporter expression 24 hrs post transduction by flow cytometry. Numbers in parentheses indicate percentage of GFP positive cells. **D.** Cell killing assay in BHK, BHK-CAR and BHK-CD46 cells transduced with Ad5/35-tk and treated with GCV for 4 days. Cytotoxicity was analyzed by the MTT assay. Error bars represent SEM. Statistics: C-D, *p*<0.01 by 2-way ANOVA.

To evaluate whether CD46 expression levels correlate with transduction of an adenoviral vector utilizing CD46 as an attachment receptor, we generated a chimeric Ad5/35-GFP virus, in which the fiber knob domain of the Ad5 capsid was replaced with the fiber knob of the species B Ad35. To characterize the transduction efficiency of the Ad5/35-GFP virus, either parental baby hamster kidney (BHK) cells, or BHK cells ectopically overexpressing CAR or CD46 were used [13]. High expression levels of CD46 in the BHK-CD46 cells were confirmed by Western blot (Figure 1A) and flow cytometric analyses (Figure 1B). Minor bands shown in WT- and CAR-BHK cells seem to result from antibody cross-reaction. To determine whether there is a correlation between CD46 and efficiency of gene transduction, the BHK cells were infected with Ad5/35-GFP using multiplicity of infection (MOI) from 5 to 100. GFP-positive cells were monitored 24 hrs post infection by fluorescence microscopy (Supplementary Figure S2A), or by flow cytometry. There was a significant dose-dependent increase in the transduction efficiency of BHK-CD46 cells compared to either wild type or BHK-CAR cells (*p*<0.01 by 2-way ANOVA) (Figure 1C). We further tested the correlation between the cytotoxic effect of the Ad5/35 virus and CD46 expression using Ad5/35-tk in combination with GCV. Cells were infected with Ad5/35-tk (5-20 MOI) and treated with GCV (10-250 μg/ml). MTT assays performed after four days of GCV treatment revealed that reduction in cell proliferation was greater in BHK-CD46 cells compared to wild type and BHK-CAR cells in an MOI dependent manner (*p*<0.01 by 2-way ANOVA) (Figure 1D). Most notably, there was an almost 80 % reduction in BHK-CD46 cell proliferation at 20 MOI and 100 μg/ml GCV (18.02 ± 3.20) compared to a 15-18 % reduction in both control cells (81.59 ± 2.95 for BHK-WT cells, 84.91±3.44 for BHK-CAR cells) (*p*<0.01). These results suggest that CD46 expression in BHK cells promotes Ad35 fiber knob mediated viral gene transduction and its viral killing efficacy.

### Ad5/35 efficiently transduces gene expression in colon cancer cells

**Figure 2:**
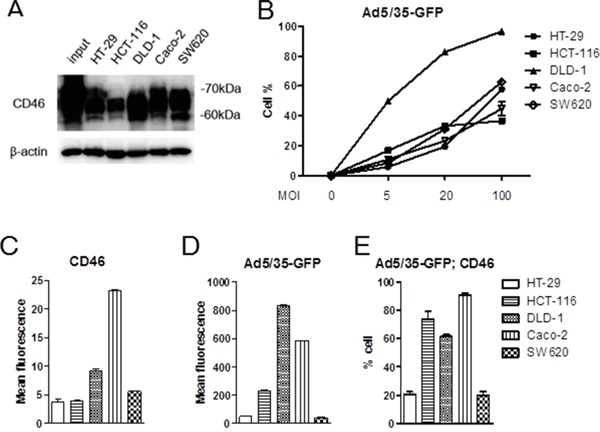
CD46 expression analysis and Ad5/35-mediated gene transduction in colorectal cancer cell lines **A.** CD46 expression in colon cancer cell lines was analyzed by Western blot analysis. **B.** CD46-positive cells were detected by flow cytometry in response to different dose of Ad5/35-GFP. **C-E.** Colon cancer cells were applied to flow cytometry to demonstrate intensity of CD46 expression (C) or Ad5/35-mediated reporter transduction (D). Percentage of cells double-stained with CD46 and GFP was shown in **E.** Error bars represent SEM. Statistics: B, *p*<0.01 by 1-way ANOVA and post-hoc Tukey test; **C-D**, *p*=0.267 by 2-way ANOVA.

To date, the role of CD46 in species B adenoviral gene transfer in CRC has not been thoroughly documented. According to Silver et al. [33], CD46 was abundantly expressed in all CRC cells. However these cells revealed variable transduction efficiencies when using species B Ad11 which binds to both CD46 and Dsg2. When we analyzed CD46 expression levels in five commonly used CRC cell lines, including HT-29, HCT-116, DLD-1, Caco-2, and SW620 by Western immunoblotting, all cells expressed abundant CD46 (Figure 2A). When cells were used for adenoviral transduction with Ad5/35-GFP, most cells responded to viral gene transduction in a dose-responsive manner, especially DLD-1 (*p*<0.01) (Figure 2B). Compared to HT-29, Caco-2, and SW620 cells, majority of DLD-1 and HCT-116 were highly responsive to viral transduction (post-hoc Tukey test). To determine the correlation between the expression level of CD46 and Ad5/35-mediated gene transduction, two color flow cytometric studies were performed using RFP for CD46 (Figure 2C) and GFP for or Ad5/35-GFP-mediated reporter expression (Figure 2D). When positive cells were demonstrated based on the mean fluorescence, CD46 level and viral gene transduction efficiency were almost equivalent, showing that there was no difference between CD46 expression level and adenoviral GFP transduction among cells (*p*=0.267 by 2-way ANOVA). When double positive cells were compared based on cell percentage, CD46 expression correlated well with adenoviral gene transduction in most CRC cells except HCT-116 (Pearson's correlation coefficient r=0.586, *p*=0.022)(Figure 2E). In HCT-116 cells, Ad5/35-mediated gene transduction was high despite the low to medium level of CD46 expression. High reporter expression was also confirmed by fluorescence microscopy (Supplementary Figure S2B). This suggests that in HCT-116 cells the level of Ad5/35-mediated gene expression was influenced by additional factors like secondary integrin receptors or by enhanced cell-specific promoter activity. However, our results were consistent with a previous report, which demonstrated that Ad11 induced more cytotoxicity in DLD-1, HCT-116, and HT-29 cells than in SW620 cells [34].

**Figure 3:**
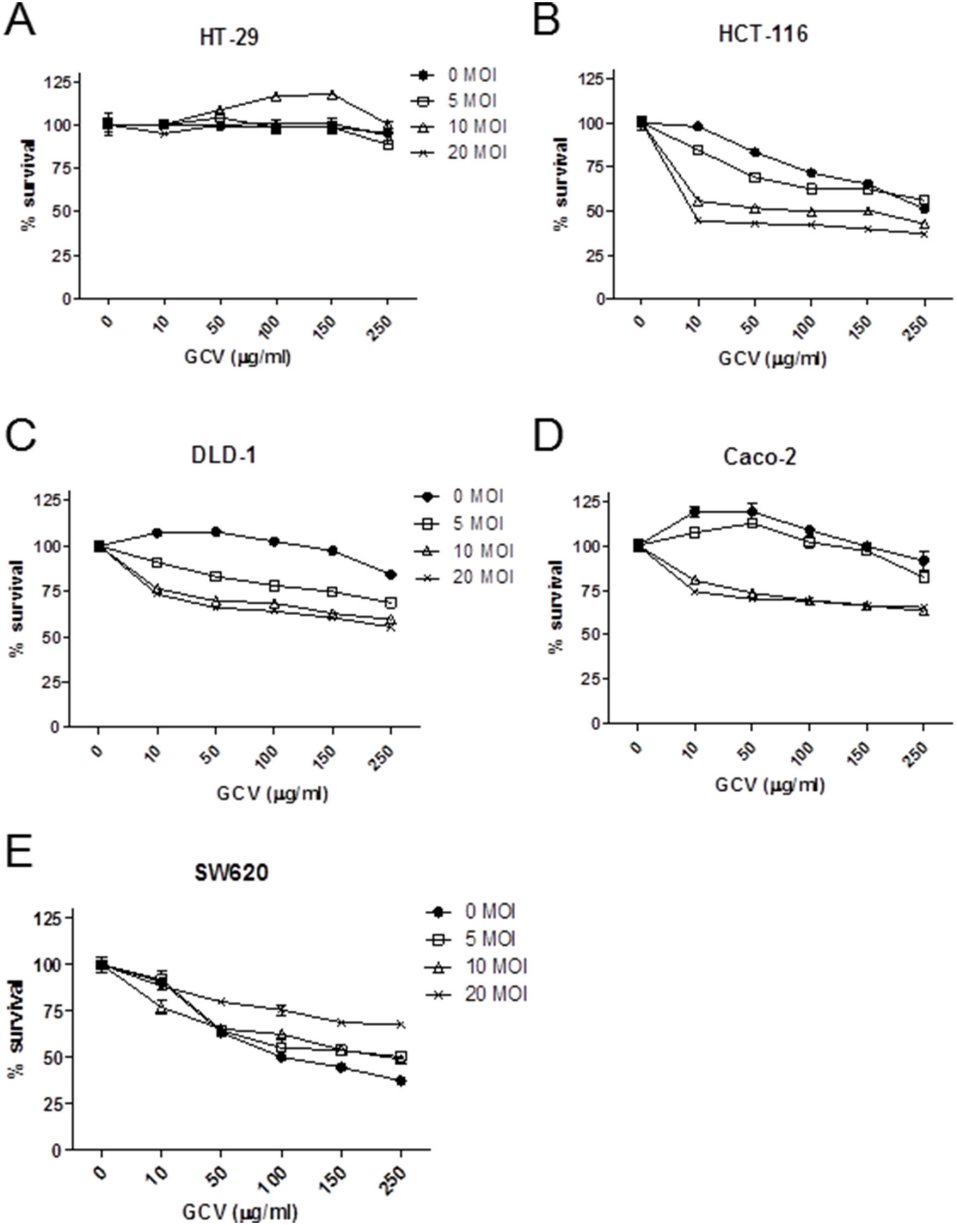
Ad5/35-tk-mediated enhancement of *in vitro* cytotoxicity in CRC cells Cells were seeded onto 24-well plates and incubated overnight. Subsequently, cells were transduced with Ad5/35-tk at the indicated MOIs ranging from 0 to 20. Varying concentrations of GCV were added the next day and MTT *in vitro* proliferation assays were performed 5 days post infection. DLD-1, Caco-2, and HCT-116 cells more effectively responded to the cytotoxic effect of Ad5/35/GCV than HT-29 and SW620 cells. Error bars represent SEM. Statistics: *p*<0.01 by 2-way ANOVA.

To link the Ad5/35-mediated gene transduction efficiency to viral cytotoxicity, the five CRC cell lines were treated with Ad5/35-tk in combination with GCV followed by cell proliferation assays 5 days post infection. As shown in Figure 3, cytotoxic effects induced by these treatments were significantly greater in HCT-116, DLD-1, and Caco-2 cells than in HT-29 and SW620 cells (*p*<0.01 by 2-way ANOVA). These results were consistent with the CD46 expression levels and virus-mediated gene transduction efficiencies shown in Figure 2C and 2D, respectively.

### CD46 promotes suicide gene therapeutics against human cancer cells *in vivo*

**Figure 4:**
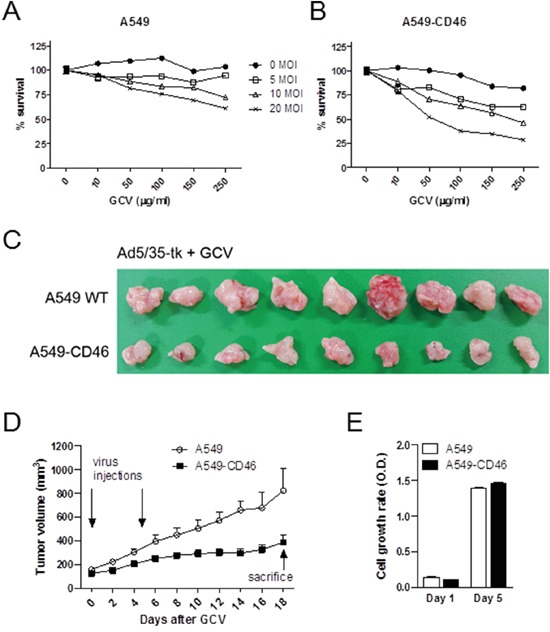
CD46 promotes Ad5/35-tk-mediated cytotoxicity for tumor growth *in vivo* **A-B.** Cells were transduced with Ad5/35-tk followed by GCV treatment. MTT *in vitro* proliferation assays were performed 5 days post infection. **C-D.** Cells were injected subcutaneously into nude mice (10-11 mice per group). Intra-tumor injections of Ad5/35-tk viruses (1.5 × 10^8^ PFU) were made twice at the indicated time points followed by GCV injections (75 mg/kg) intraperitoneally on days 2–12. Tumor growth was measured by a caliper at the indicated time points. **E.** Cell proliferation of parental A549 and CD46-overexpressing A549 was measured by the MTT assay. Error bars represent SEM. Statistics: A-B, *p*<0.01 by 2-way ANOVA; D, *p*=0.005 by repeated-measures ANOVA; E, *p*=0.44 by 1-way ANOVA.

To test the *in vivo* cell killing efficacy of CD46-utilizing Ad5/35-tk, CD46-overexpressing human A549 human lung adenocarcinoma cells and M010119 melanoma cells were used. In these cells, endogenous CD46 expression levels were further increased following transduction with a lentiviral expression construct resulting in about 2-fold increase for A549 cells, and 9-fold increase for M010119 cells [35](also in unpublished data). CD46 overexpression in these cells was confirmed by Western blot (Supplementary Figure S3A). By the MTT proliferation assay, treatment with Ad5/35-tk plus GCV more effectively killed CD46-overexpressing A549 cells compared to wild type cells (*p*<0.01 by 2-way ANOVA) (Figure 4A–4B). There was a more than 50 % reduction in A549-CD46 cell proliferation at 20 MOI and 100 μg/ml GCV (37.66 ± 3.02) compared to that in control cells (75.74 ± 3.11). To further confirm the therapeutic effect of CD46-utilizing Ad5/35-tk/GCV on tumor growth *in vivo*, CD46-overexpressing A549 and parental A549 cells were injected subcutaneously into the backs of nude mice. When tumor volumes reached around 150 mm^3^, 1.5 × 10^8^ plaque-forming units (PFU) of Ad5/35-tk were injected intratumorally on days 1 and 6. Then, 75 mg/kg of GCV was administered intraperitoneally on days 2–12. Compared to the control group, the CD46-A549 cells-injected group showed a dramatic decrease in tumor volume at 18 days after virus inoculation (*p*=0.005 by repeated-measures ANOVA) (Figure 4C–4D). Average tumor volume in the CD46-A549 cells-injected group was 387.33 ± 57.83 mm^3^, whereas average tumor volume in the wild type A549 cells-injected group was 821.39 ± 191.3 mm^3^. In contrast, cell culture growth rates of CD46-A549 and control cells were not altered significantly without the treatments (*p*=0.440) (Figure 4E). Ad5/35-tk in combination with GCV was similarly effective in CD46-overexpressing M010119 melanoma cells both *in vitro* and *in vivo* (Supplementary Figure S3B-D).

### CD46 overexpression indicates better survival of patients with CRC

**Figure 5:**
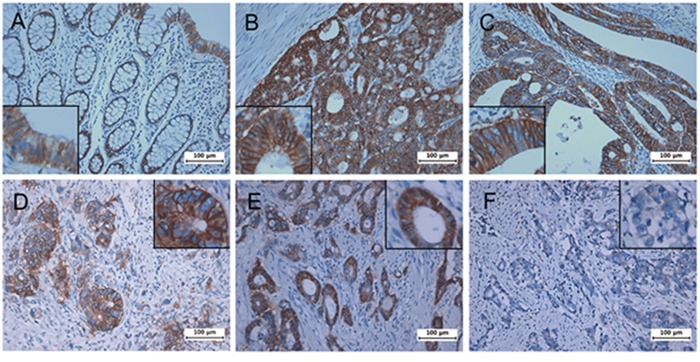
Immunohistochemical analysis of CD46 expression in patient colorectal cancers Tumors were analyzed for the expression of CD46 by immunostaining. **A,** normal mucosa. **B-C,** well-differentiated tumors. **D-F,** poorly differentiated and highly invasive tumors. While CD46 was generally overexpressed in colorectal tumors compared to normal counterparts, some invasive cancers showed no expression of CD46. High-powered pictures are shown in inlets of selected areas.

**Figure 6:**
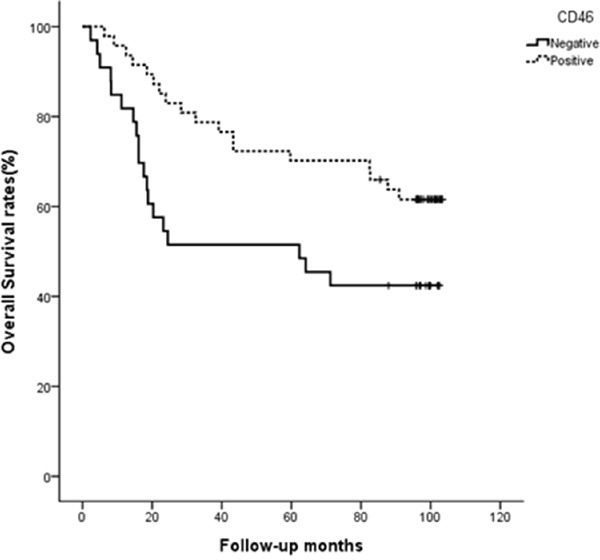
CD46 expression indicates better survival of CRC patients Overall survivability was demonstrated by Kaplan-Meier curve and measured by the log-rank test (*p*=0.031).

To investigate on a possible correlation between CD46 expression and clinico-pathological features in patients with CRC, eighty CRC samples from patients with a full clinical record were evaluated by immunohistochemistry. Samples were scored by two investigators including a pathologist. While the expression of CD46 in the normal columnar mucosal epithelium of the colon was weak (Figure 5A), CD46 was very highly expressed in both well-differentiated (Figure 5B–5C) and poorly differentiated CRCs (Figure 5D–5E). Immunostaining pattern of CD46 was mostly membranous in both normal and cancerous cells. Majority of poorly differentiated CRCs with a highly invasive phenotype did not express CD46 (Figure 5F). When CD46 expression of score 2-3 was arbitrarily set as positive expression, CD46 expression was positively correlated with differentiation (*p*=0.041) and negatively correlated with perineural invasion (*p*=0.038), tumor stage (*p*=0.003), and distant metastasis (*p*=0.001) (Table 2). In addition, the overall survivability of CRC patients with positive CD46 expression was significantly higher than that of CRC patients with negative CD46 expression (χ^2^=4.678, *p*=0.031 by the log-rank test) (Figure 6). These results suggest that CD46 expression is highly maintained in well-differentiated CRC cells and it is lost in poorly differentiated aggressive tumor cells.

**Table 2:** Correlation between CD46 expression and clinico-pathological features in patients with colorectal cancers

	Total (n=80)	CD46		p-value
		Negative (n=33)	Positive (n=47)	
**Age (years)**				0.089
< 66.6	37	19	18	
≥ 66.6	43	14	29	
**Sex**				0.404
Male	48	18	30	
Female	32	15	17	
**Tumor size (cm)**				0.841
< 4.6	45	19	26	
≥ 4.6	35	14	21	
**Histologic type**				0.041
Differentiated	72	27	45	
Undifferentiated	8	6	2	
**Lymphovascular invasion**				0.536
Negative	54	21	33	
Positive	26	12	14	
**Perineural invasion**				0.038
Negative	54	18	36	
Positive	26	15	11	
**Stage**				0.003
I / II	40	10	30	
III / IV	40	23	17	
**Depth of invasion (T)**				0.96
T1 / T2	24	10	14	
T3 / T4	56	23	33	
**Lymph node metastasis (N)**				0.362
N 0	3	2	1	
N 1-3	77	31	46	
**Distant metastasis**				0.001
M 0	61	19	42	
M 1	19	14	5	

Pearson's χ2 test was used

## DISCUSSION

CD46 is a cell-surface regulatory protein that prevents lysis of autologous human cells by activated complement and is considered to be ubiquitously expressed. It has also been demonstrated that CD46 is implicated in the development and progression of several cancers. CD46 expression is elevated in medulloblastoma [32], while CD46 expression has been linked with poor prognosis in breast cancer [30]. It has been proposed that overexpression of CD46 may serve to protect cancer cells from complement-dependent cytotoxicity, thereby evading destruction caused by the immune system [36, 37]. Our study demonstrated that CD46 is generally highly expressed in colorectal cancers, and based on this phenomenon, species B-based adenoviral gene therapy may be better suited than the currently used species C adenoviral vector like Ad5. Ad5-based viral therapies have several shortcomings, including downregulation of its receptor in cancer cells. In fact, many tumor cells express relatively low levels of Ad5 receptors, including CAR [4, 5, 38]. Our tumor microarray data also showed that a low level of CD46 expression in normal liver which may imply that adenoviral targeting of CD46 may results in liver toxicity. However, Ad11, another group B adenovirus, induced significantly lower liver toxicity in animals in comparison to Ad5, maybe due to the lack of interaction of Ad11 with coagulation factor X and the consequent absence of hepatocyte infection [39, 40]. Therefore, chimeric Ad5 vectors that are capable of targeting CD46 hold the promise to be more efficient gene delivery vehicles than Ad5 vectors with native fibers. In order to show that CD46 plays decisive role in species B adenoviral gene therapy for CD46-overexpressing colon cancers, we developed chimeric Ad5/35 vectors with fiber knobs from Ad35 to specifically target CD46 in cancer cells.

Generally, CD46 expression was higher in most cancer cells compared to CAR expression [34]. In this study, we demonstrate that Ad5/35 more efficiently delivers GFP and cytotoxic genes to CD46-expressing rodent cells compared to CD46 negative parental cells or CAR-expressing control cells. The same phenomenon was confirmed in various colon cancer cells, where CD46 expression levels were found to correlate with viral gene transduction and cytotoxic effects. Thus, while CD46 was ubiquitously expressed in all five colon cancer cells used in this study, more efficient Ad5/35-mediated gene transduction was achieved in HCT-116, DLD-1, and Caco-2 cells compared to HT-29 and SW620 cells. Cell toxicity and proliferation assays also indicated that Ad5/35-tk in combination with GCV had the highest cell-killing activity in HCT-116, DLD-1, and Caco-2 cells. However, in a study using replication competent Ad11, HT-29 cells showed high viral transduction and viral cytotoxicity compared to other CRC cells including HCT-8 and LS174T [33]. Among the CRC cells, HT-29 cells were more sensitive to Ad11, while SW620, HCT-116, and DLD-1 cells were more sensitive to Ad5 [34]. This may be due to the presence of other species B adenoviral receptors such as desmoglein 2 and receptor X [15, 41]. Many cancer cells that originated from the pancreas, prostate, breast, colon, ovary, and lung were also Ad11-sensitive [34]. These included Hs766T, Capan-1, Capan-2, PaTu 8988s, PC-3, MCF7, MDA-MB-468, SK-BR-3, and HT-29. Our *in vivo* experiments showed significant growth inhibition of ectopically CD46-overexpressing A549 lung cancer and M010119 melanoma cells in xenograft mice treated with Ad5/35-tk in combination with GCV compared to parental cells. Thus, CD46 enhances the cytotoxic effect of species B adenoviral gene therapy. In fact, treatment of A549 cells with CD46 siRNA resulted in a decrease of transduction with Ad3-EGFP [12].

CD46 expression was closely analyzed and compared to clinico-pathological parameters, and CD46 upregulation was observed in highly differentiated, locally confined, and non-metastasized CRC. Expression of CD46 also implies better survivability of patients with CRC. It has been reported that CD46 expression was significantly higher in colon cancer tissues compared with adjacent normal colon tissues. While CD46 was found to have no clinical relevance, levels of CD55 and CD59, other complement inhibitor membrane cofactor proteins, were positively correlated with the differentiation and tumor stage in colon cancers [31]. Breast cancers are reported to consistently express CD46. Using tissue microarray, strong CD46 expression was exhibited by 27% of the breast tumors. CD46 expression was significantly associated with tumor grade, histological type of tumor, and tumor recurrence, but there was no correlation with lymph node stage or the presence of vascular invasion. Poor-prognosis tumors that lose CD55 and CD59 still express CD46. It has been suggested that loss of CD55 and CD59 could be compensated by expression of CD46 [21]. Expression of CD46 also represents an independent risk factor for disease-free survival and overall survival, demonstrating that patients with tumors negative for CD46 have an increased progression-free time and overall survival time as compared with patients with CD46-positive tumors. A study demonstrates that breast cancers manifest CD46 expression and that it is linked to a less favorable prognosis [30].

In this study, we show that CD46 is highly expressed in most colorectal tumors, when compared to matching normal mucosa. This makes a strong case that colorectal cancers represent good targets for CD46-targeting species B adenovirus-mediated gene therapy. In fact, chimeric Ad5/35 vectors targeting CD46 are known to be better tools than vectors targeting CAR for cancer targeted gene therapy. While most colon cancer cells express substantial amounts of CD46, samples from cancer patients show that there is an inverse correlation between CD46 expression and clinico-pathological parameters in terms of undifferentiation, invasion, metastasis, T stage, and survival, suggesting that adenoviral gene therapy may not be equally effective in patients with highly advanced colorectal cancers. Therefore, suitability of species B adenoviral gene therapy in certain cancer types may need careful patient-specific adjustments.

## MATERIALS AND METHODS

### Patient samples and immunohistochemistry

Multi-tumor tissue microarray slides (TARP2) were obtained from the Cooperative Human Tissue Network under the Tissue Array Research Program (TARP) of the National Cancer Institute, The National Institutes of Health (Bethesda, MD, USA). This study also included eighty colorectal cancer specimens with normal matching tissues from patients who provided informed consent at Chonnam National University Hospital. All cases had a clinical follow-up for at least 10 years. Immunohistochemistry was performed as previously described [42]. Briefly, colorectal tumor tissues were deparaffinized and rehydrated in graded alcohols and distilled water. Antigens were retrieved by heating the tissues in a microwave for 5 min in 10 mM sodium citrate buffer (pH 6.0). Endogenous peroxidase activity was destroyed with 3% H_2_O_2_ in methanol for 30 min. After blocking nonspecific sites, tissues were incubated overnight at 4°C with anti-CD46 antibody (1:200, Origene, Rockville, MD, USA). The slides were then incubated with biotinylated anti-rabbit antibody (1:400) for 30 min at room temperature. Subsequently, the signals were amplified by the horseradish peroxidase-DAB detection method (Vector Laboratories, Inc., Burlingame, CA, USA). The sections were counterstained with hematoxylin for microscopic evaluation. The stained slides were evaluated by two different investigators, including a pathologist, who were blinded to the patient's clinical features. The intensity of staining was classified into one of two grades: positive (medium to strong staining) or negative (no to low and focal staining).

### Viruses

To construct Ad5/35CMV-GFP, pAd1020sfidA-CMV-GFP, which was described previously [43], was digested with the restriction enzyme *Sfi*I to release CMV-GFP-pA and the ψ packaging signal. The fragment encompassing the CMV-GFP-pA and the ψ packaging signal was recombined with RightZap1.3 (OD260, Boise, ID, USA) in HEK293 cells. The virus particles were purified by CsCl_2_ gradient ultracentrifugation. To construct Ad5/35CMV-tk, the GFP gene was replaced by the thymidine kinase gene (tk).

### Cell culture

Human colon cancer cell lines, including HT-29, HCT-116, DLD-1, Caco-2, and SW620 were purchased from American Type Culture Collection (ATCC, Manassas, VA, USA). Baby hamster kidney (BHK) cells expressing CAR and CD46, and A549 human non-small cell lung cancer cells and A549 cells overexpressing CD46, as well as CD46-overexpressing M010119 human melanoma cells were described previously [13, 35]. All cells were routinely cultured in Dulbecco's modified Eagle's medium media (DMEM, Invitrogen, CA, USA) supplemented with 10% heat-inactivated fetal bovine serum (FBS, Invitrogen, CA, USA) at 37°C in an atmosphere containing 5% CO_2_. All cultures were fed with fresh medium every 3-4 days.

### Western blotting

Cells were grown to 80% confluence and were lysed in lysis buffer (50 mM Tris-HCl, 150 mM NaCl, 1% NP-40, 0.5% DOC and protease inhibitors). Twenty μg of total cell lysates were loaded onto 10% SDS-polyacrylamide gel and separated using a Bio-Rad electroporation system. After the proteins were transferred to a Immobilon–P membrane (Millipore, Billerica, MA, USA), the membranes were blocked with 5% nonfat dry milk in Tris-buffered saline containing 0.1% Tween-20 (TBST) at room temperature for 1 hr. The membrane was further incubated overnight with primary antibodies. Polyclonal rabbit anti-β-actin antibodies were obtained from Santa Cruz Biotechnology Inc. (Santa Cruz, CA, USA) and rabbit monoclonal CD46 antibody was obtained from OriGene Technologies, Inc. (Rockville, MD, USA). After washing with TBST, the membrane was incubated for 1 hr with horseradish peroxidase-conjugated secondary antibodies. The blots were developed using Immobilon Western detection system (Millipore, Billerica, MA, USA). Signals were detected and analyzed by using a LAS4000 luminescent image analyzer (Fuji, Tokyo, Japan).

### FACScan analysis

Cells were grown to 80% confluence in 100 mm culture dishes. Cells were harvested and incubated with rabbit polyclonal CD46 antibody (Origene, Rockville, MD, USA) for 30 min at 4°C. After washing, cells were further incubated with Alexa 488-labeled anti-rabbit IgG (Invitrogen, Carlsbad, CA, USA). Then, cells were analyzed by flow cytometry using a FACScan (BD Biosciences, San Jose, CA, USA). For viral infectivity assays, cells were seeded into 60 mm culture dishes and incubated with Ad5/35-GFP virus at different multiplicities of infection (MOI 0, 5, 20, 100) at 37°C. After 3 hrs, viruses were removed by replacing with fresh media. At 24 hrs after infection, cells were harvested and washed with PBS. Then, cells were analyzed by flow cytometry. Results were analyzed by Kaluza Analysis Software (Beckman Coulter, Inc. Brea, CA, USA)

### Cell killing assay

For evaluating the *in vitro* cell killing efficacy of Ad5/35- thymidine kinase (Ad5/35-tk) in combination of Ganciclovir (GCV, JHP Pharmaceuticals LLC., MI, USA), cells were seeded into a 24-well plate at 1 × 10^4^ cells per well. The next day, media were changed to 500 μl of DMEM/ BSA (20 mg/ml) containing Ad5/35-tk with various MOI doses (5, 10, 20). At 24 hrs post infection, increasing concentrations (0, 10, 50, 100, 150, and 250 μg/ml) of GCV were added to the wells for 4 days. Cell viability was assessed by the MTT assay. Briefly, 50 μl of 3-(4,5-dimethylthiazol-2-yl)-2,5-diphenyltetrazolium bromide (MTT) solution (0.5 mg/ml) was added to the cells and cells were incubated for 4 hrs. The absorbance was measured at 570 nm using a microplate reader with SOFTmax PRO software (Molecular Devices, Sunnyvale, CA, USA).

### Immunofluorescence

Cells were grown on glass coverslips in 12-well plates. Cells were incubated with Ad5/35-GFP virus up to 100 MOI at 37°C. At 3 hrs after infection, cells were changed to fresh media. At 24 hrs after infection, cells were washed three times with PBS. Coverslips were mounted with Prolong Gold anti-fade reagent. Photographic images were acquired by using IX51 fluorescence microscope (Olympus, Tokyo, Japan).

### *In vivo* killing assays

Female nude mice (6 weeks old) were obtained from Central Lab. Animal Inc. (Korea). All experiments were performed in accordance with our institution's guidelines for animal care. Mice were housed and monitored under constant humidity and temperature. Parental A549 or A549-GFP-CD46 (5 × 10^6^) cells were subcutaneously injected into the flank of mice. When tumor volume reached around 150 mm^3^, 1.5 × 10^8^ plaque-forming units (PFU) of Ad5/35-tk were injected intratumorally on days 1 and 6. Then, 75 mg/kg of GCV was administered intraperitoneally on days 2–12. Tumor size was measured every 2 days and tumor volume was calculated using the following equation: length × width^2^ × 0.5.

### Statistical analysis

All data were expressed as mean ± standard error (SEM) and processed using the SPSS 21.0 software system (IBM, Chicago, IL). Viral efficacy comparisons among the cells were tested with either one-way or two-way ANOVA. Tumor cytotoxicity studies using tumor-bearing mice were analyzed by repeated-measures ANOVA. Correlation between CD46 expression and various clinico-pathological parameters was compared using the Pearson's χ^2^ test and Fisher's exact test. Kaplan-Meier method with the log-rank test was applied to demonstrate survival. Statistical significance was achieved when *p* was less than 0.05.

## SUPPLEMENTARY FIGURES AND TABLES


