# Supplementary material for: Efficacy of CD46-targeting chimeric Ad5/35 adenoviral gene therapy for colorectal cancers
Source: Oncotarget. 2016 May 18;7(25):38210–23. doi: 10.18632/oncotarget.9427 (PMC5122383; doi:10.18632/oncotarget.9427)
Supplement: Supplementary file 1 [file oncotarget-07-38210-s001.pdf]

SUPPLEMENTARY FIGURES

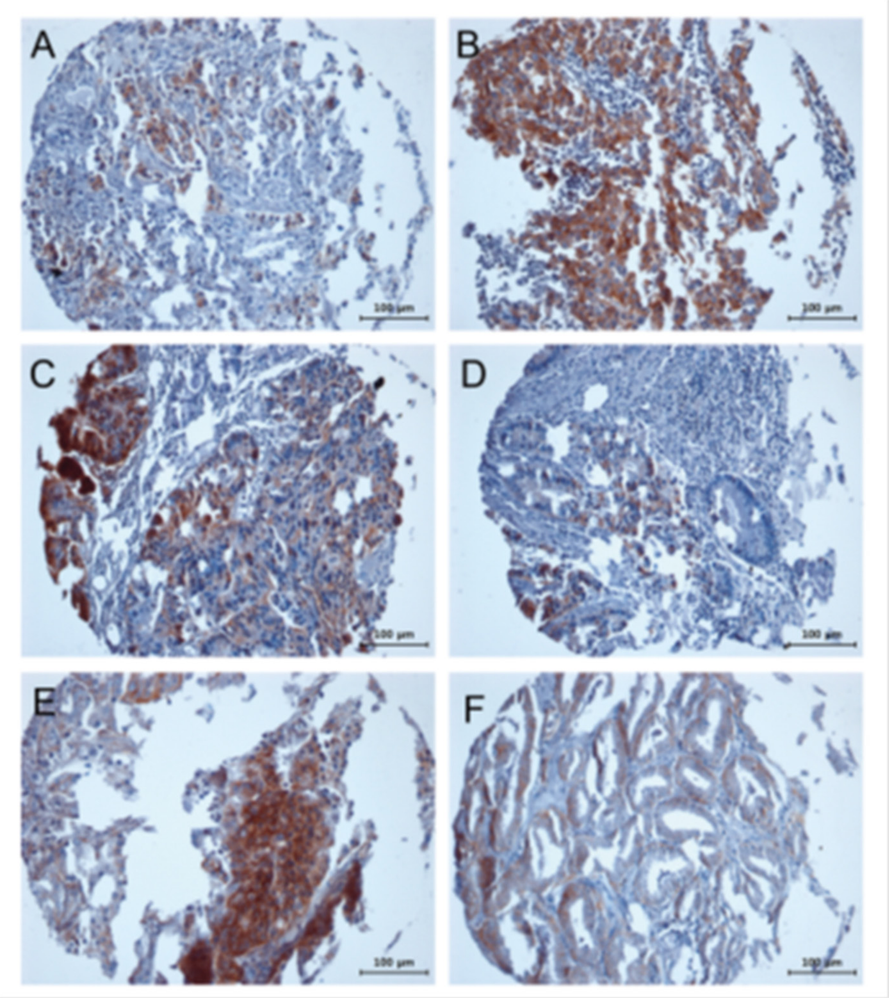

Supplementary Figure S1: Expression of CD46 in tumor microarray. **A.** breast cancer; **B.** lung cancer; **C-D.** colorectal cancer; **E-F.** prostate cancer.

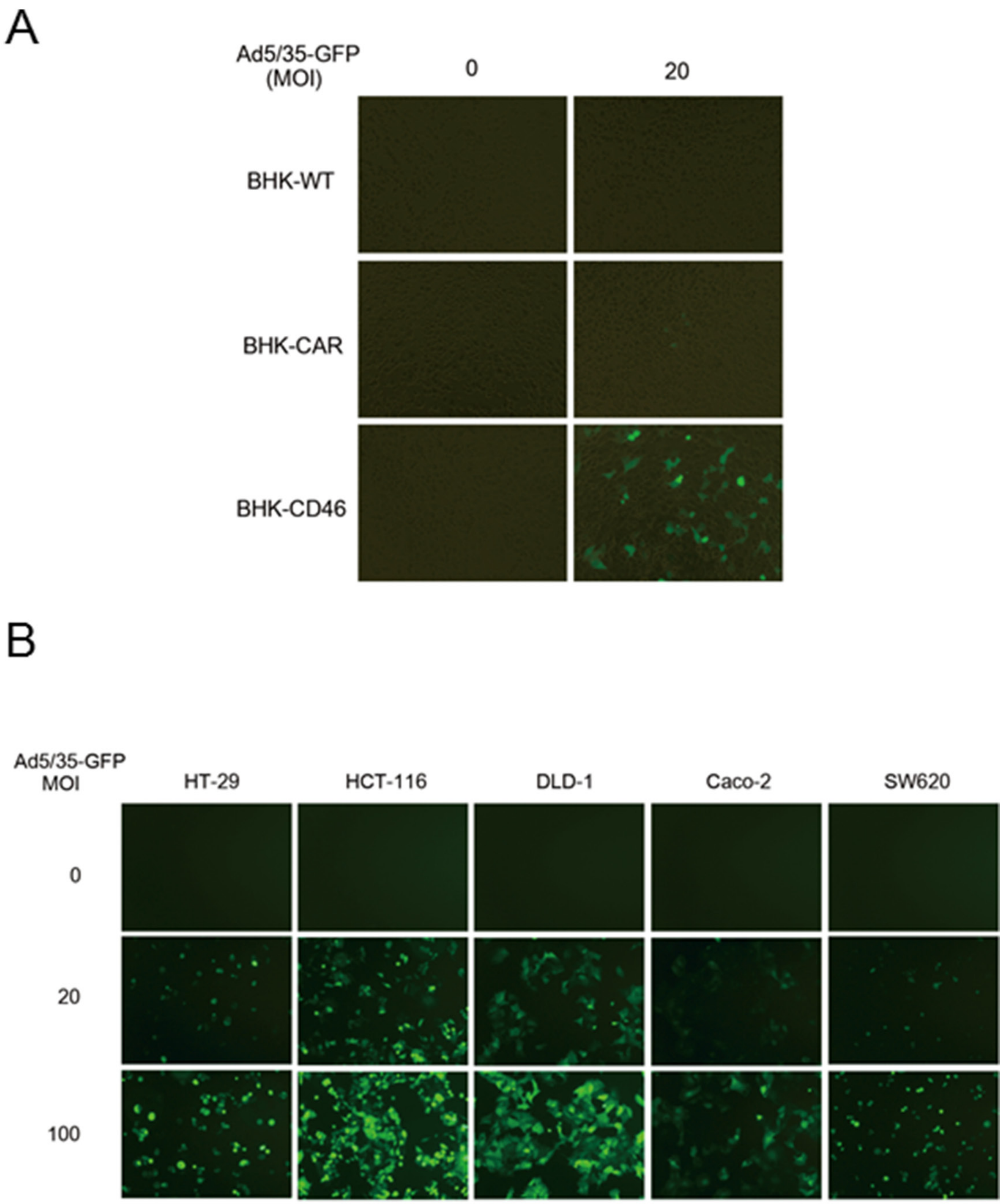

Supplementary Figure S2: Ad5/35-mediated GFP transduction in various cells. Either rodent parental, CAR, and CD46-expressing BHK cells **(A)** or five human colon cancer cell lines **(B)** were infected with the indicated concentrations of Ad5/35-GFP. At 24 hrs post infection GFP expression levels were monitored by fluorescence microscopy (20X).

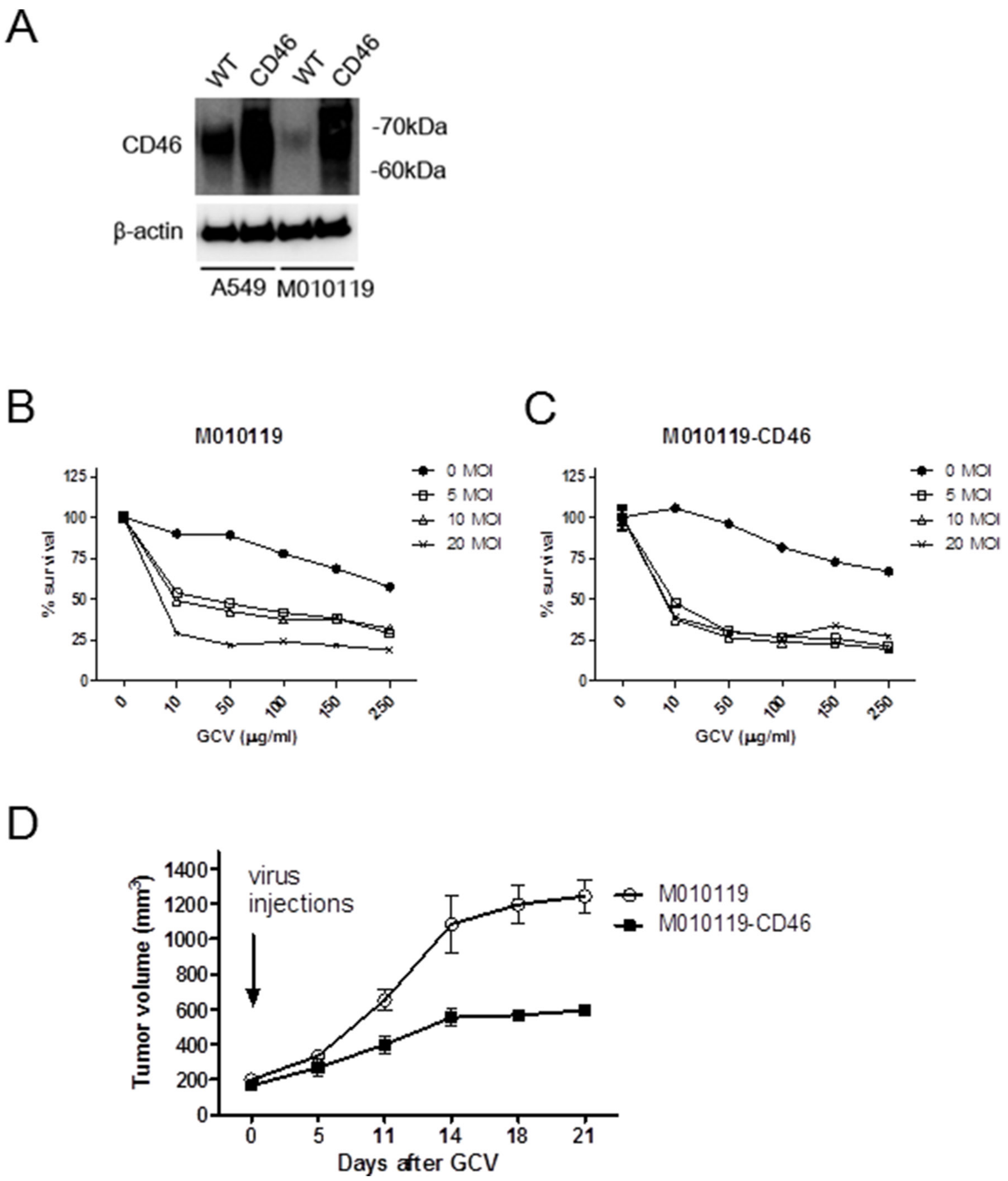

Supplementary Figure S3: CD46 promotes Ad5/35-mediated cytotoxicity for M010119 tumor growth *in vivo*. A. Western blotting analysis of CD46 expression in A549 lung cancer and M010119 melanoma cells. Increased CD46 expression over endogenous levels was achieved by lentiviral transduction in A549 and M010119 cells. **B-C.** Parental M010119 and CD46-overexpressed M010119 cells were transduced with Ad5/35-tk followed by GCV treatment. MTT *in vitro* proliferation assays were performed 5 days post infection. **D.** Parental M010119 and CD46-overexpressed M010119 cells were injected subcutaneously into nude mice. Intra-tumor injections of Ad5/35-tk were made at the indicated time points followed by intraperitoneal GCV injections on days 2–15. Tumor growth was measured by a caliper at the indicated time points ($N_{M010119}$=3, $N_{M010119\text{-}CD46}$=3). Error bars represent SEM. Statistics: B-C, p=0.013 by 2-way ANOVA; D, p=0.014 by repeated-measures ANOVA.
